# Interval-cohort designs and bias in the estimation of per-protocol effects: a simulation study

**DOI:** 10.1186/s13063-019-3577-z

**Published:** 2019-09-05

**Authors:** Jessica G. Young, Rajet Vatsa, Eleanor J. Murray, Miguel A. Hernán

**Affiliations:** 1 Department of Population Medicine, Harvard Medical School & Harvard Pilgrim Health Care Institute, Boston, USA; 2 Pathways M.D. Program, Harvard Medical School, Boston, USA; 3 Department of Epidemiology, Harvard T.H. Chan School of Public Health, Boston, USA; 4 Department of Biostatistics, Harvard T.H. Chan School of Public Health, Boston, USA; 5 Harvard-MIT Division of Health Sciences and Technology, Boston, USA; 6 Department of Epidemiology, Boston University School of Public Health, Boston, USA

**Keywords:** Per-protocol effect, Inverse probability weighting, Interval cohorts, Simulation

## Abstract

**Background:**

Randomized trials are considered the gold standard for making inferences about the causal effects of treatments. However, when protocol deviations occur, the baseline randomization of the trial is no longer sufficient to ensure unbiased estimation of the per-protocol effect: post-randomization, time-varying confounders must be sufficiently measured and adjusted for in the analysis. Given the historical emphasis on intention-to-treat effects in randomized trials, measurement of post-randomization confounders is typically infrequent. This may induce bias in estimates of the per-protocol effect, even using methods such as inverse probability weighting, which appropriately account for time-varying confounders affected by past treatment.

**Methods/design:**

In order to concretely illustrate the potential magnitude of bias due to infrequent measurement of time-varying covariates, we simulated data from a very large trial with a survival outcome and time-varying confounding affected by past treatment. We generated the data such that the true underlying per-protocol effect is null and under varying degrees of confounding (strong, moderate, weak). In the simulated data, we estimated per-protocol survival curves and associated contrasts using inverse probability weighting under monthly measurement of the time-varying covariates (which constituted complete measurement in our simulation), yearly measurement, as well as 3- and 6-month intervals.

**Results:**

Using inverse probability weighting, we were able to recover the true null under the complete measurement scenario no matter the strength of confounding. Under yearly measurement intervals, the estimate of the per-protocol effect diverged from the null; inverse probability weighted estimates of the per-protocol 5-year risk ratio based on yearly measurement were 1.19, 1.12, and 1.03 under strong, moderate, and weak confounding, respectively. Bias decreased with measurement interval length. Under all scenarios, inverse probability weighted estimators were considerably less biased than a naive estimator that ignored time-varying confounding completely.

**Conclusions:**

Bias that arises from interval measurement designs highlights the need for planning in the design of randomized trials for collection of time-varying covariate data. This may come from more frequent in-person measurement or external sources (e.g., electronic medical record data). Such planning will provide improved estimates of the per-protocol effect through the use of methods that appropriately adjust for time-varying confounders.

**Electronic supplementary material:**

The online version of this article (10.1186/s13063-019-3577-z) contains supplementary material, which is available to authorized users.

## Background

In randomized trials, the *per-protocol effect* is the effect that would have been estimated if all participants had adhered to their randomly assigned treatment strategies during the entire follow-up [1]. However, because adherence to the assigned treatment strategy is not in itself randomized, a naive comparison that excludes trial participants who fail to adhere to their assigned strategies will generally be biased [2].

For example, in a trial of a new treatment versus standard of care to treat coronary heart disease, adherers to the treatment may be individuals who also tend to take antihypertensive treatment. Thus, a lower rate of disease among adherers may simply reflect their higher uptake of antihypertensives rather than a benefit of the treatment under study. Therefore, analyses that attempt to estimate the per-protocol effect typically need to adjust for prognostic factors that, like antihypertensive use in our example, are also associated with adherence. That is, per-protocol analyses are observational analyses of the randomized trial data and therefore need to adjust for confounders.

In randomized trials of point interventions that are administered shortly after randomization (e.g., a one-dose vaccination, a one-time screening test), adherence to the assigned intervention is fully determined at baseline and therefore can only be affected by baseline factors. The implication is that per-protocol analyses of point interventions only need to adjust for baseline confounders. On the other hand, in randomized trials of treatment strategies that are sustained during the follow-up (e.g., treatment for coronary heart disease, antiretroviral treatment for HIV-positive patients), adherence to the treatment strategy must also be sustained during the follow-up. The implication of this potentially time-varying adherence is that per-protocol analyses of sustained strategies need to adjust for time-varying confounders — time-varying prognostic factors that affect treatment decisions — as well as for baseline confounders — baseline prognostic factors that affect treatment decisions [3–6]. For example, in a randomized trial to estimate the effect of two antiretroviral therapies on mortality, an increased alcohol intake during the follow-up is a time-varying confounder because it affects both the risk of death and of non-adherence to the assigned treatment.

It follows that valid estimation of the per-protocol effect of sustained treatment strategies requires adequate data collection of treatment and confounders after randomization. Many randomized trials collect such post-randomization data, but most only do so at pre-specified intervals (e.g., every 12 months). Because non-adherence may occur at any time during the follow-up, the confounders measured at the pre-specified times may not be sufficient or relevant to adjust for non-adherence that took place at an unknown time between the pre-specified measurement times.

In this paper, we review the impact of interval measurement on the estimation of per-protocol effects in randomized trials [7]. We conduct a simulation study to illustrate the potential magnitude of bias, even using causal inference methods for longitudinal settings such as inverse probability (IP) weighting [8], which appropriately account for time-varying confounders affected by past treatment.

## Methods

### Simulation design

We simulated data from a hypothetical randomized trial to quantify the effect of a new drug treatment compared to the standard of care on 5-year mortality risk.

Each individual is assigned to either the new drug treatment (*Z*=1) or to standard of care (*Z*=0) and followed until death or the administrative end of the study (60 months post-randomization), whichever comes first. We assume the exact month of death is known, as is common when studies link their data with death registries. For simplicity and without loss of generality, no individual is lost to follow-up.

**Fig. 1:**
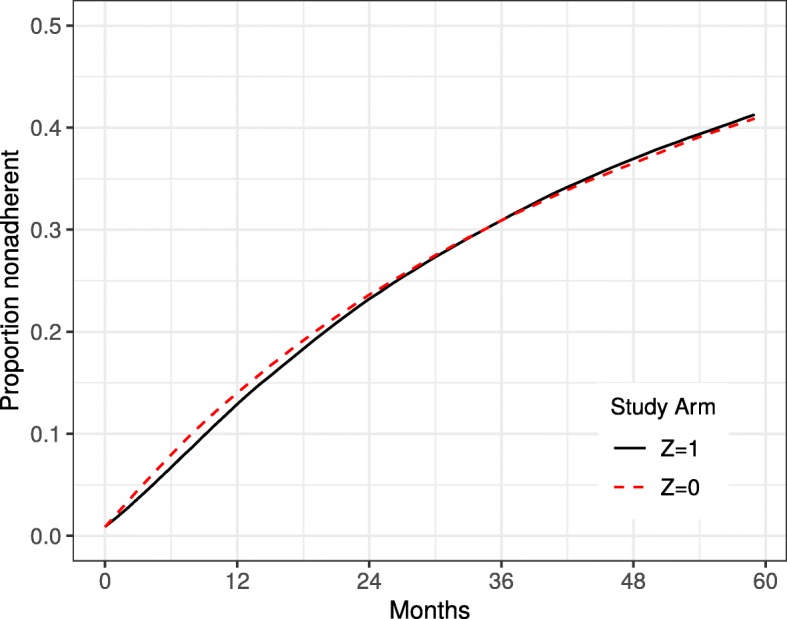
Proportion of participants who deviate from the protocol over the study period by treatment arm

Define *t*=0,…,60 as an index of follow-up month with *t*=0 the month of randomization (baseline). Let *Y*_*t*_ be an indicator of death by month *t* with *Y*_0_≡0 for all individuals (all participants are alive and therefore at risk of the outcome at baseline) and *A*_*t*_ an indicator of whether the new drug treatment is taken in month *t*. An individual deviated from the protocol in the first month *t* in which *A*_*t*_≠*Z*. In our simulated study approximately 40% of individuals in both arms deviated from the protocol at some point during the follow-up. Figure 1 shows the cumulative proportion of protocol deviations over the study period by treatment arm.

In randomized trials, treatment *A*_*t*_ will typically depend on both baseline (e.g., sex, race, baseline age) and post-baseline (e.g., lab measurements, concomitant medications) risk factors for the outcome (e.g., death). Let *L*_*t*_=(*L*_1*t*_,*L*_2*t*_) be a vector of such risk factors in month *t*, with *L*_1*t*_ a lab measurement (continuous) and *L*_2*t*_ the use of a concomitant medication (binary).

**Fig. 2:**
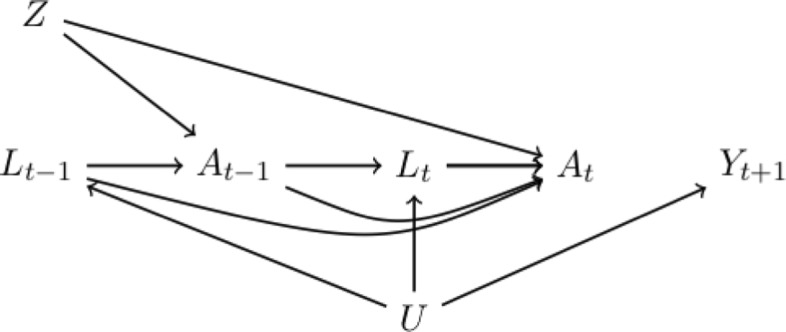
A causal diagram representing the underlying mechanism for protocol deviations in our study

The causal diagram in Fig. 2 outlines the data-generating process of our simulated study. The node *U* represents a vector of baseline unmeasured outcome risk factors that also may affect *L*_*t*_ (e.g., genetic factors) with no direct effect on treatment at any time (as depicted by the absence of an arrow from *U* into *A*_*t*−1_ or *A*_*t*_ in Fig. 2). As expected in many realistic settings, the time-varying covariates *L*_*t*_ also may be affected by past treatment adherence (as depicted by the arrow from *A*_*t*−1_ to *L*_*t*_ in Fig. 2). For example, adherence to the standard versus the new treatment may affect values of future lab measurements.

We generated the data such that 100,000 individuals are assigned to each arm. We quantified bias for a given approach by the difference between the effect estimate obtained by that approach in this very large sample and the true effect value. Had we used a smaller sample size (e.g., 100 individuals assigned to each arm), random variability could explain some differences between effect estimates and the true values of the effect (unless we had used the average over a large number of small samples, which is nearly equivalent to generating a single very large sample — this is illustrated in Additional file 2).

We generated the data such that both the causal effect of treatment *A*_*t*_ for all *t* and the direct effect of randomization (*Z*) not mediated through treatment are null, as shown in Fig. 2 by the absence of any *causal paths* (paths consisting of arrows going in the same direction) connecting *Z*, *A*_*t*−1_, or *A*_*t*_ with the future outcome (*Y*_*t*+1_). Therefore, both the intention-to-treat effect and the per-protocol effect are null.

### Data-generating models

We generated longitudinal data according to the following models for each subject *i*=1,…,200,000 (*i*=1,…,100,000 assigned *Z*_*i*_=1 and *i*=100,001…,200,000 assigned *Z*_*i*_=0): *U*_*i*_ was generated from a uniform distribution between 0 and 1. Then the following were generated for each month *t*=0 until *t*=59 or until *Y*_*t*+1*i*_=1 was generated, whichever came first:

- *L*_1*ti*_ was generated from a normal distribution such that $ L_{1ti}=6U_{i}-A_{t-1i}-\text {cumavg}(\overline {A}_{t-2i})+0.25\text {cumavg}(\overline {L}_{1t-1i})+0.01t+\epsilon _{i}$ with *ε*_*i*_∼*N*(0,*σ*=2), $\text {cumavg}(\overline {A}_{t-2i})$ is the cumulative average of (*A*_0*i*_,…,*A*_*t*−2*i*_), and $\text {cumavg}(\overline {L}_{1t-1i})$ is the cumulative average of (*L*_10*i*_,…,*L*_1*t*−1*i*_).
- *L*_2*ti*_ was generated from a Bernoulli distribution with mean *p*_*L*2*i*_, equal to the probability that *L*_2*t*_=1 given individual *i* ’s treatment and covariate history and survival to *t*, defined such that $\text {logit} (p_{L2i})=-5+3U_{i}+1.25\text {cumavg}(\overline {L} _{1ti})+0.5L_{2t-1i}+0.25A_{t-1i}+0.25\text {cumavg}(\overline {A} _{t-2i})+0.01t$.
- For any individual *i* deviating from the protocol by *t*−1 (i.e., *A*_*t*−1*i*_≠*Z*_*i*_), we set *A*_*ti*_=*A*_*t*−1*i*_ (once an individual stops complying we assume they stay non-compliant). Alternatively, for any individual *i* complying with the protocol through *t*−1 (i.e., all *A*_*ji*_=*Z*_*i*_ for *j*<*t*), *A*_*ti*_ was generated from a Bernoulli distribution with mean *p*_*Ai*_, equal to the probability that *A*_*t*_=1 given individual *i*’s treatment and covariate history and survival to *t*, such that
  1 $$ {}\text{logit}(p_{Ai})=\alpha_{0}+0.4\text{cumavg}(\overline{L} _{1ti})+0.35L_{2t-1i}.   $$
  For individuals assigned *Z*_*i*_=1 (active treatment), we set *α*_0_=4.0. For individuals assigned *Z*_*i*_=0 (standard of care), we set *α*_0_=−6.5.
- The death indicator *Y*_*t*+1*i*_ was generated from a Bernoulli distribution with mean *p*_*Yi*_, equal to the probability that *Y*_*t*+1_=1 given individual *i*’s treatment and covariate history and survival to *t*, such that
  2 $$ \text{logit}(p_{Yi})=\theta_{0}+\theta_{1}U_{i}.   $$

We considered three versions of this data-generating mechanism, varying the values of *θ*_0_ and *θ*_1_ in the model (2). As we explain in the section Defining and estimating the per-protocol effect, given our data-generating models, the magnitude of *θ*_1_ determines the magnitude of time-varying confounding (and *θ*_0_ the baseline event rate). We considered the following variations: “strong confounding” *θ*_1_=8(*θ*_0_=−11), “moderate confounding” *θ*_1_=3(*θ*_0_=−7), and “weak confounding” *θ*_1_=0.5(*θ*_0_=−6). We also considered three variations of the “strong confounding” scenario under different choices of *α*_0_ in model (1) that reduced the chance of deviating from the protocol in both arms. Table 1 displays the cumulative proportion of protocol deviations by the end of the study period by treatment arm resulting from different choices of *α*_0_.

**Table 1:** Proportion of protocol deviations under different choices of *α*_0_ in (1) by arm under “strong confounding”

Scenario	Arm	*α*_0_	Cumulative proportion deviated	
0	*Z*=1	4.0	41%	
	*Z*=0	-6.5	41%	
1	*Z*=1	5.0	21%	
	*Z*=0	-7.5	20%	
2	*Z*=1	6.0	9%	
	*Z*=0	-8.5	8%	

R code implementing this simulation design is provided in Additional file 1.

### Defining and estimating the intention-to-treat effect

We can define the intention-to-treat effect for any follow-up month *t*+1=1,…,60 as a contrast of the cumulative risks in arm *Z*=1, Pr[*Y*_*t*+1_=1|*Z*=1] versus in arm *Z*=0, Pr[*Y*_*t*+1_=1|*Z*=0]. Our data generation, under all scenarios, is consistent with no confounding for the effect of *Z* on survival, as illustrated in Fig. 2 by the absence of any *open backdoor paths* (open paths consisting of arrows going in different directions and, therefore, non-causal paths) [9] connecting the treatment arm indicator *Z* and the future outcome *Y*_*t*+1_. As a result, and because of the absence of loss to follow-up, a simple comparison of the estimated risks (i.e., cumulative incidences) in arm *Z*=1 versus arm *Z*=0 is an unbiased estimator of the intention-to-treat effect Pr[*Y*_*t*+1_=1|*Z*=1] versus Pr[*Y*_*t*+1_=1|*Z*=0] at any post-randomization time *t*+1=1,…,60.

We are able to recover the true intention-to-treat effect in our study, regardless of the presence of protocol deviations, because unbiased estimation of the intention-to-treat effect only relies on the random assignment of *Z* and no loss to follow-up. In contrast, unbiased estimation of the *per-protocol effect* requires additional assumptions.

### Defining and estimating the per-protocol effect

Let $Y_{t+1}^{\overline {a}=\overline {1}}$ denote an individual’s indicator of death by month *t*+1, had she, possibly contrary to fact, continuously followed the protocol in arm *Z*=1. Similarly, let $Y_{t+1}^{\overline {a}= \overline {0}}$ denote this outcome by month *t*+1, had she, instead, continuously followed the protocol in arm *Z*=0. We can then formally define the per-protocol effect at month *t*+1 as a contrast of the *counterfactual* risks:

3 $$ \Pr \left[Y_{t+1}^{\overline{a}=\overline{1}}=1|Z=1\right]\text{ versus} \Pr \left[Y_{t+1}^{ \overline{a}=\overline{0}}=1|Z=0\right].   $$

Note that, because *Z* was randomly assigned, we could alternatively define the per-protocol contrast as $\Pr \left [Y_{t+1}^{\overline {a}=\overline {1}}=1\right ] \text {versus} \Pr \left [Y_{t+1}^{\overline {a}=\overline {0}}=1\right ]$ (unconditional on *Z*). Many randomized trials include a “naive” per-protocol analysis in which the survival curves are estimated after censoring participants at the time that they deviate from the protocol. This “naive” approach generally fails to recover the true per-protocol effect because it fails to account for confounding for the effect of received treatment due to risk factors that affect both future adherence and survival. In Fig. 2, such confounding is represented by open backdoor paths connecting *A*_*t*−1_ and *A*_*t*_ to *Y*_*t*+1_, e.g., the path *A*_*t*_←*L*_*t*_←*U*→*Y*_*t*+1_. The data-generating models we have described previously ensure the presence of this path by the dependence of *A*_*t*_ on past values of the time-varying risk factors (*L*_0_,…,*L*_*t*_), the dependence of *L*_*t*_ on *U*, and the dependence of *Y*_*t*+1_ on *U*. As described in the section “Data-generating models”, we varied the degree of confounding (strong, moderate, or weak) by varying the magnitude of the parameter *θ*_1_ in the model (2), which quantifies the strength of the dependence of *Y*_*t*+1_ on *U*.

Even though there is confounding for the per-protocol effect, the data generation mechanism in our study still allows unbiased estimation of the per-protocol effect as long as the study actually recorded all monthly covariates *L*_*t*_ and treatments *A*_*t*_. Graphically, in Fig. 2 there are no open backdoor paths connecting *A*_*t*−1_ and *A*_*t*_ to *Y*_*t*+1_*conditional* on past time-varying covariate changes [9]. For example, the open backdoor path *A*_*t*_←*L*_*t*_←*U*→*Y*_*t*+1_ is blocked by conditioning on *L*_*t*_. Note that the measurement of the variable *U* is unnecessary to adjust for confounding when the variables *L*_*t*_ are measured in all *t*.

However, valid estimation of the per-protocol effect (3) requires the use of adjustment methods that, like IP weighting, can handle the fact that *L*_*t*_ is affected by past treatment [3, 4, 10]. We give a detailed description of the IP weighting algorithm in Additional file 2 and the R code in Additional file 1. Briefly, this approach involves: (1) as in the naive analysis, censoring participants when they deviate from their assigned protocol; (2) estimating IP weights which, at each time, are either 0 for censored participants or the reciprocal of the cumulative product of the time-varying probabilities of adherence to the protocol given the participant’s measured confounder history up to that time for uncensored participants; and (3) estimating IP weighted survival curves. Risk differences and risk ratios can then be estimated by the complement of the IP weighted survival estimates. In addition to full measurement of the time-varying covariates, the validity of this approach also relies on correct specification of the model for the adherence probabilities in step 2.

### Estimating the per-protocol effect under interval measurement

In practice, many randomized trials are conducted as interval cohorts such that adherence and covariates are recorded only at regular, scheduled follow-up times. When there are gaps between measurement times, the full history of treatment and covariate changes over the follow-up will not be completely observed and, generally, there will be unmeasured confounding; that is, under our data-generating assumption represented by Fig. 2, open backdoor paths will remain after conditioning on only the measured past. Also, the full history of treatment changes will be only partially observed. Under a non-null scenario, failure to measure interim treatment changes may produce an additional source of unmeasured confounding for treatment effects even at measured times; e.g., in Fig. 2, were there an arrow from *A*_*t*−1_ into *Y*_*t*+1_, then an unblockable open backdoor path (by failure to measure *A*_*t*−1_) connecting *A*_*t*_ and *Y*_*t*+1_ would be present. Partial knowledge of treatment changes thus also requires some form of imputation to estimate the per-protocol effect which is defined by counterfactual intervention in all months, not only months in which measurements are taken. Any imputation method may rest on strong assumptions, for example, imputation under the assumption that treatment does not change during measurement gaps or under missing at random (MAR) assumptions [11].

Suppose, without loss of generality, that the interval between measurements is constant throughout the follow-up, e.g., *m* months. We computed an IP weighted estimator of the per-protocol effect (3) and corresponding estimates of the counterfactual survival curves had all participants continuously complied with the protocol in each treatment arm under an interval-cohort scenario with *m*=12, that is, a scenario in which treatment and covariate changes are measured only at baseline and then every 12 months. In interim months, treatment and covariates were set to the last measured value and the contribution to the weight cumulative product set to 1 for all subjects at these times. In this scenario, there will be residual confounding by failure to adjust for time-varying covariates at unmeasured times. At measured times, IP weights can only be based on the inverse probability that a subject continues to adhere in month *s* given her partially measured confounder history. This probability is unknown under our data-generating mechanism (because we generated each *A*_*t*_ from the full history). Thus, we would also expect some bias due to model misspecification under this scenario. Here we chose to model adherence based on the cumulative average of past measured values of the continuous time-varying covariate (based on only the baseline and every 12-month measurement) and the current value of the binary covariate (as the value from the previous month, the true value needed, will not be measured in this case).

## Results

### Intention-to-treat effect estimates

**Fig. 3:**
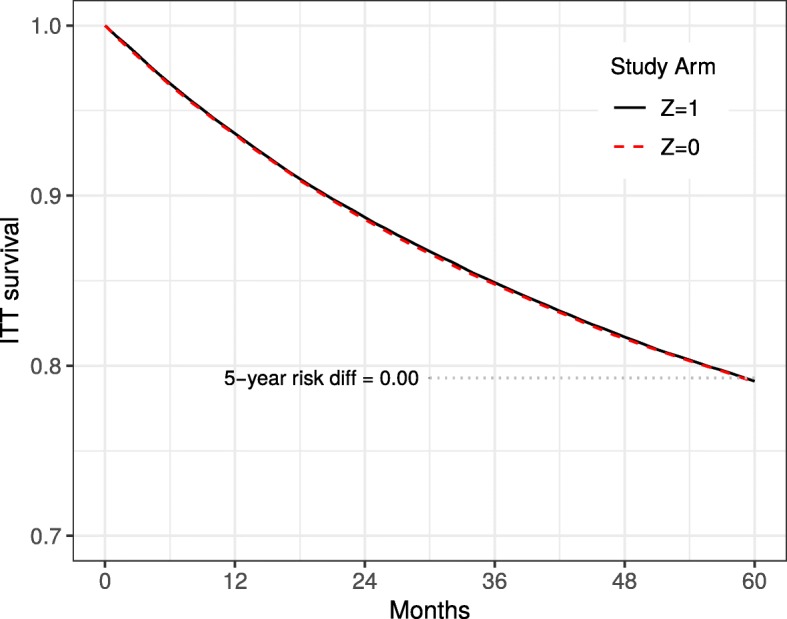
Intention-to-treat survival estimates by treatment arm

Figure 3 shows the estimated intention-to-treat survival curves Pr[*Y*_*t*+1_=0|*Z*=1] and Pr[*Y*_*t*+1_=0|*Z*=0] based on the the cumulative proportion of deaths in each arm by each follow-up month. Results are shown for the “strong confounding” scenario and the main study of approximately 40% deviators per arm (Scenario 0 in Table 1). As expected, there is no bias in these estimates of the intention-to-treat effect; the curves completely overlap, which is consistent with the fact that the true intention-to-treat effect is null in all months *t*+1.

### Naive versus IP weighted per-protocol effect estimates under full measurement

**Fig. 4:**
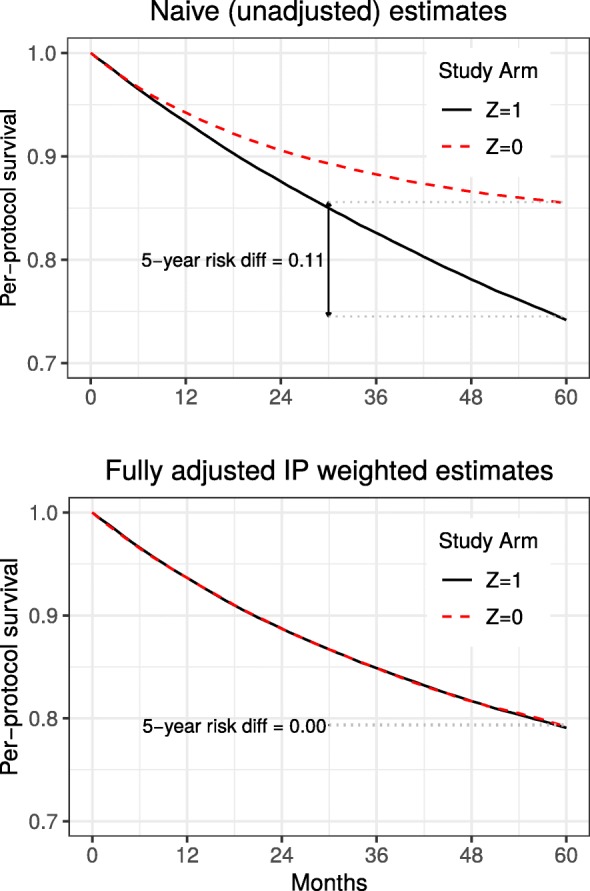
Naive versus IP weighted estimates under strong confounding but complete measurement of covariate history

As illustrated by the top panel of Fig. 4, in our study a “naive” unweighted estimator that ignores time-varying confounders fails to recover the true null per-protocol effect because the curves do not overlap. Rather the estimates of the per-protocol 5-year risk difference and risk ratio for standard versus new treatment are 0.11 and 1.77, respectively. The bottom panel of Fig. 4 shows IP weighted estimates of the per-protocol effect under full measurement of the time-varying covariates (*m*=0). As expected, the estimated survival curves completely overlap, consistent with the truth, which is null. Figure 4 depicts results only under strong confounding. As expected, survival estimates across treatment arms under the naive approach that ignores confounding become closer as the strength of confounding weakens, while IP weighted estimates of the survival curves completely overlap under all scenarios (weak and moderate results are not shown).

### IP weighted per-protocol effect estimates under interval measurement

**Fig. 5:**
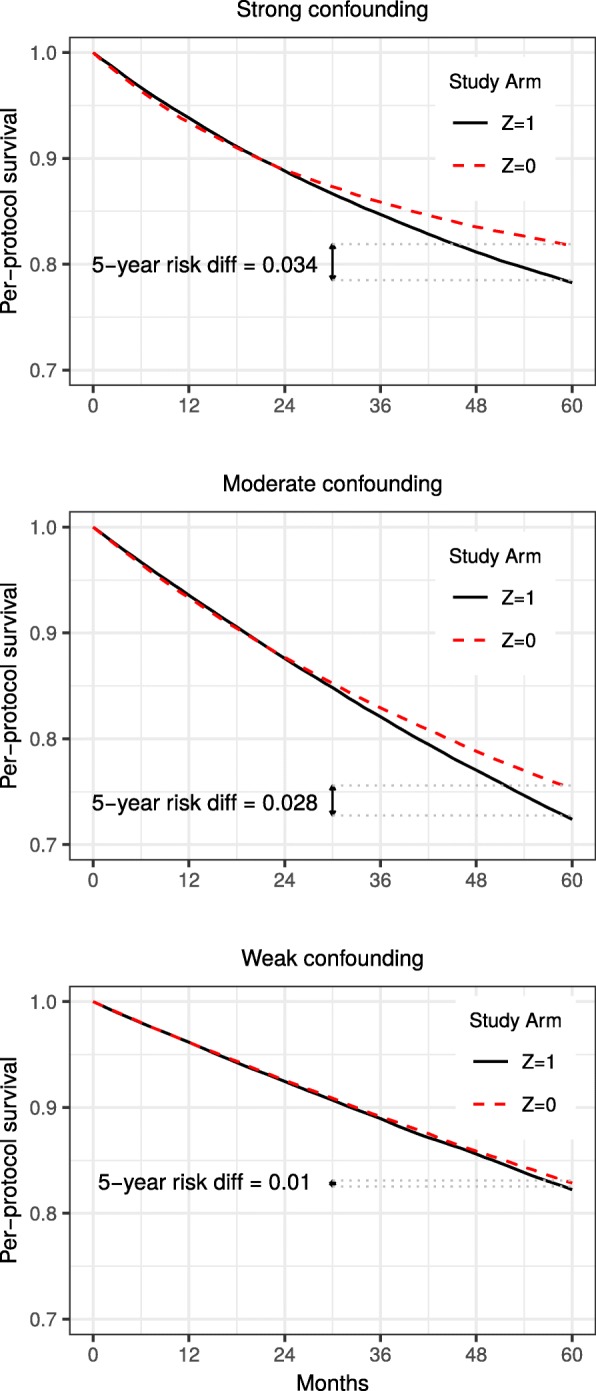
IP weighted estimates of per-protocol survival under the *m*=12 interval-measurement scenario and different confounding scenarios

In the interval-measurement scenario, we are generally unable to recover the truth of no per-protocol effect. In our study, IP weighted per-protocol effect estimates under *m*=12 diverged from the null as the strength of confounding increased. Specifically, Fig. 5 shows that differences in the survival curves increase with the strength of confounding, which results in 5-year risk difference/risk ratio estimates of 0.034/1.19 under strong confounding, 0.028/1.12 under moderate confounding, and 0.01/1.03 under weak confounding in our large sample.

**Fig. 6:**
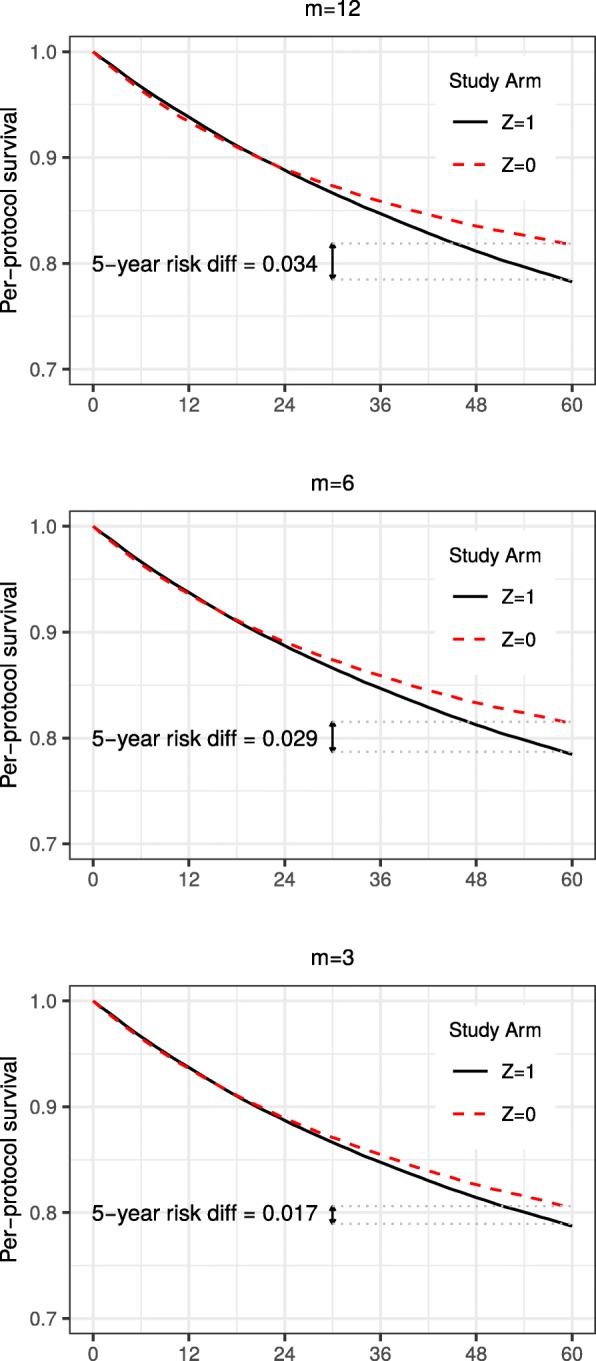
IP weighted estimates of per-protocol survival under strong confounding and decreasing values of *m*

Figure 6 illustrates that, even under strong confounding, bias decreases with more frequent measurement; estimates of the 5-year risk difference get closer to the truth of zero with decreasing *m*. Specifically, the IP weighted estimates of the risk difference/risk ratio were 0.017/1.02 under *m*=3,0.029/1.04 under *m*=6, and 0.034/1.19 under *m*=12.

**Fig. 7:**
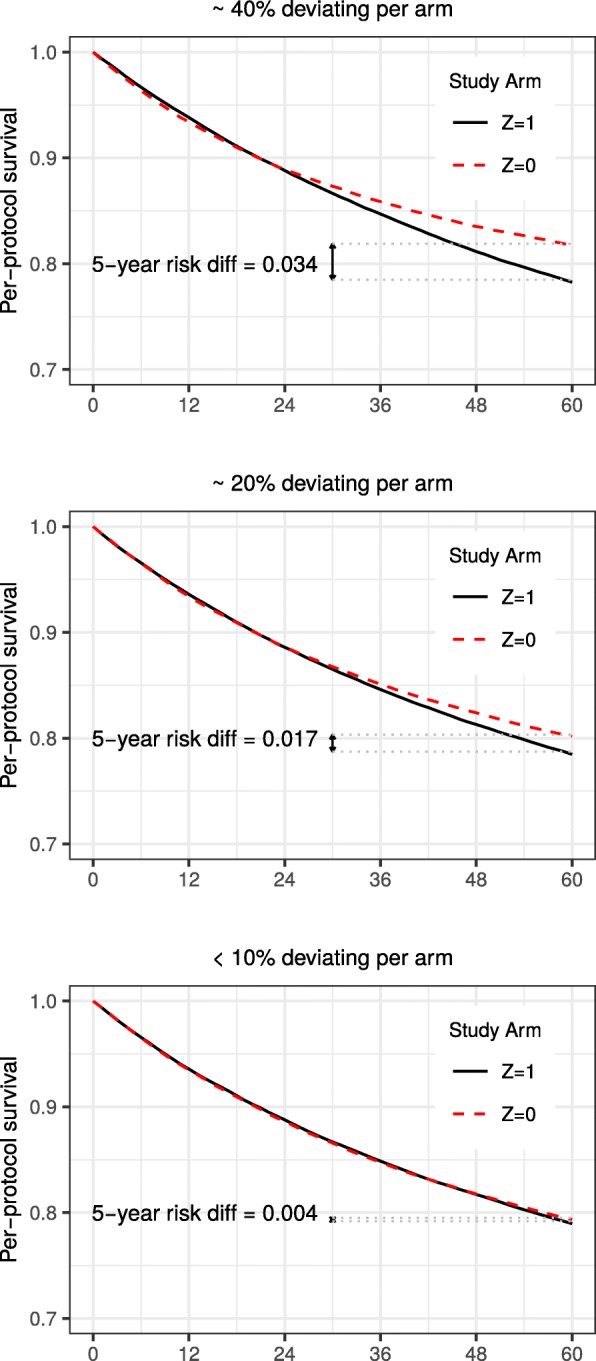
IP weighted estimates of per-protocol survival under strong confounding and decreasing proportion deviating

Finally, Fig. 7 illustrates that, even under strong confounding and long interval measurement (*m*=12), bias diminishes with decreasing non-adherence. Specifically, when the proportion of deviators decreased from approximately 40% (Scenario 0 in Table 1) to 20% (Scenario 1 in Table 1), the IP weighted estimates of the risk difference/risk ratio were closer to the null. Bias was negligible, with risk difference/ratio estimates of 0.004/1.005, when there were fewer than 10% deviators per arm (Scenario 2 in Table 1).

## Discussion

We used a simulation to study bias in the estimation of per-protocol effects in randomized trials with interval-cohort designs. Bias arose even using methods such as IP weighting, which appropriately adjust for time-varying confounders. However, IP weighted estimates were less biased than estimates from a naive analysis that ignored time-varying confounding.

We considered the simple case of per-protocol effects defined by static treatment strategies (e.g., always take the new treatment versus always take the standard treatment), but our approach could also be applied to dynamic strategies under which treatment changes in response to pre-specified events (e.g., a drug toxicity) [12–14]. Also, we considered a simulation without censoring by loss to follow-up. Censoring may prevent unbiased estimation of both per-protocol and intention-to-treat effects without sufficient and appropriate adjustment for baseline and time-varying covariates [10, 15].

The bias created by interval measurement in the estimation of time-varying treatment effects has been previously highlighted in the computer science literature [16] and in epidemiological studies such as the Framingham Heart Study and the Nurses’ Health Study [7, 17]. In practice, the interval length required to make the bias negligible will depend on the frequency with which treatment and confounders can change. For example, in studies of treatments that rarely change more than once per month (like the one in our simulation), an interval length of one month will likely suffice. In other studies, measures of more frequent covariate changes may be necessary. In addition to more frequent in-person follow-up, complementary data sources such as electronic health records and pill cap monitors can help capture these changes.

## Conclusions

The bias that arises from interval measurement highlights the need for randomized trials designed to collect post-baseline data on time-varying prognostic factors and adherence. This data may be obtained from various sources (e.g., more frequent in-person follow-up, electronic health records, pill cap monitors). Such planning, aided by the use of causal diagrams representing subject matter knowledge and assumptions, will ultimately provide improved estimates of the per-protocol effect, an informative complement to the intention-to-treat effect.

## Additional files

Additional file 1 R code to implement the simulation and IP weighted estimation procedures. (R 23 kb)

Additional file 2 Technical details of the IP weighted estimation algorithm and comparison of bias calculation using a single large sample versus average of many small samples. (PDF 166 kb)

## Data Availability

Not applicable.

## Abbreviations

IP: Inverse probability
MAR: Missing at random
