# Supplementary material for: Interval-cohort designs and bias in the estimation of per-protocol effects: a simulation study
Source: Trials. 2019 Sep 5;20:552. doi: 10.1186/s13063-019-3577-z (PMC6729016; doi:10.1186/s13063-019-3577-z)
Supplement: Supplementary file 2 — Technical details of the IP weighted estimation algorithm and comparison of bias calculation using a single large sample versus average of many small samples. (PDF 166 kb) [file 13063_2019_3577_MOESM2_ESM.pdf]

Appendix "Interval-cohort designs and bias in the estimation of per-protocol effects: a simulation study"

Jessica G. Young, Rajet Vatsa, Eleanor J. Murray, Miguel A. Hernán

1 Details of inverse probability weighted estimation when full time-varying covariate history is measured

We estimated the counterfactual survival by a given month $t+1$ in arm $Z=z$ had everyone in that arm adhered to the protocol as follows in a person-time data set with one record per study participant in arm $Z=z$ per month of his/her follow-up under full data collection:

1. Artificially censor a participant the first month in which he/she deviates from the protocol (i.e. the first time $A_t \neq z$). This month of censoring will be the last record in the artificially censored data set for that subject.

2. For each record indexed by a particular subject and month $s \leq t$ in the artificially censored data, attach a weight to that record which takes the value 0 if the subject first deviated from the protocol at time s and otherwise takes the value:

$$wt(s) = \frac{\prod_{j=0}^{s} \hat{\Pr}(A_j = z|\overline{A}_{j-1} = \overline{a}_{j-1}, Y_j = 0, Z = z)}{\prod_{j=0}^{s} \hat{\Pr}(A_j = z|\overline{L}_j, \overline{A}_{j-1} = \overline{a}_{j-1}, Y_j = 0, Z = z)}$$

   where "overbars" are used to represent history of a covariate through the specified time index (e.g. $\overline{A}_{j-1}$ is observed treatment history through $j-1$), $\overline{a}_{j-1}$ is a vector of constants all equal to z, $\prod_{j=0}^{s}$ represents taking the product from month 0 through month s, $\hat{\Pr}(A_j = z|\overline{A}_{j-1} = \overline{a}_{j-1}, Y_j = 0, Z = z)$ is an estimate of the overall probability the subject adhered to the protocol in month j and $\hat{\Pr}(A_j = z|\overline{L}_j, \overline{A}_{j-1} = \overline{a}_{j-1}, Y_j = 0, Z = z)$ is an estimate of this same probability but conditional on the study participant's covariate history through j (which we denote $\overline{L}_j$). Both the numerator and denominator probabilities can be estimated from the data via pooled logistic over time regression models.

3. Estimate the hazard in each month $s = 1, \ldots, t+1$ for arm $Z = z$ that would have been observed under full adherence as $h_s(z) = \frac{\sum_{i=1}^{n_{z,s}} Y_{i,s} wt(i,s)}{\sum_{i=1}^{n_{z,s}} wt(i,s)}$ where $n_{z,s}$ are the number of subjects in arm $Z=z$ who were still alive and uncensored in the previous month $s-1$.

4. From the monthly hazards, estimate the survival probability by month $t+1$ for am $Z=z$ that would have been observed under full adherence as $S(t+1,z)=\prod_{s=1}^{t+1}(1-h_s(z))$.

The above algorithm is repeated in each arm $Z=1$ and $Z=0$ for each follow-up month $t+1=1,\ldots,60$ to obtain survival curves. An estimate of the per-protocol effect is obtained by a contrast in $1-S(1+1,z=1)$ vs. $1-S(t+1,z=0)$. This estimate will recover the true per-protocol effect under our data generating mechanism (as depicted by the causal diagram in the main text) and also provided the pooled logistic regression model used to estimate the weight denominator probabilities is correctly specified. Because we simulated A_t directly from this model, we fit this model according to the true data generating model under the full data collection scenario.

R code is provided in a separate supplementary file.

2 Comparison of bias calculations based on a single large sample versus the average of many smaller samples

Appendix Figures 1-4 show that the calculation of bias is nearly equivalent comparing estimates based on a single sample of 100,000 individuals per arm compared with the average of estimates based on many samples of either 100 or 500 individuals per arm. This is illustrated for bias under the main scenario of strong confounding and approximately 40% nonadherence per arm (scenario 0 of Table 1 in the main text) in the estimate of the intention to treat effect (Figure 1), the naive (unadjusted) estimate of the per-protocol effect (Figure 2), the IP weighted estimate of the per-protocol effect under full measurement (Figure 3) and the IP weighted estimate of the per-protocol effect under yearly interval measurement (Figure 4).

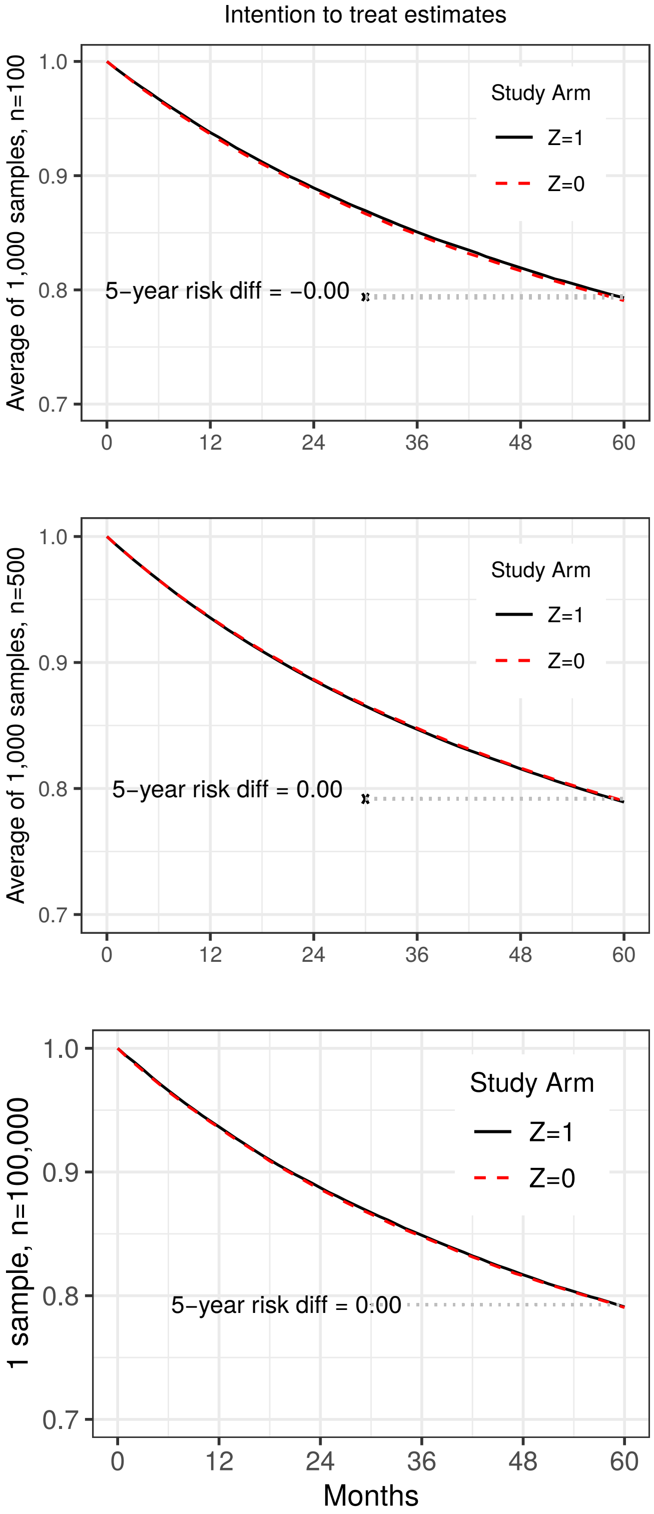

Figure 1: Intention-to-treat survival estimates by treatment arm. Comparison of bias calculations using a single large sample compared to an average of many smaller samples

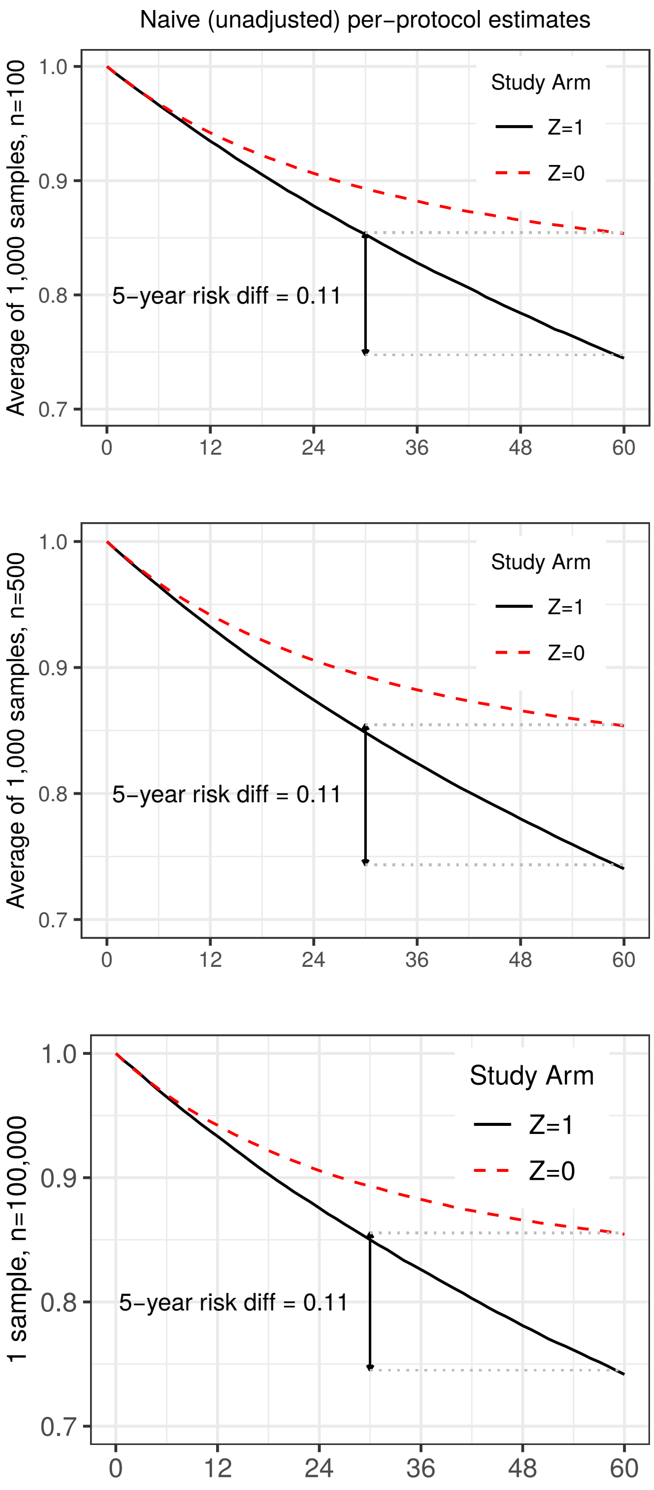

Figure 2: Naive (unadjusted) per-protocol survival estimates by treatment arm. Comparison of bias calculations using a single large sample compared to an average of many smaller samples

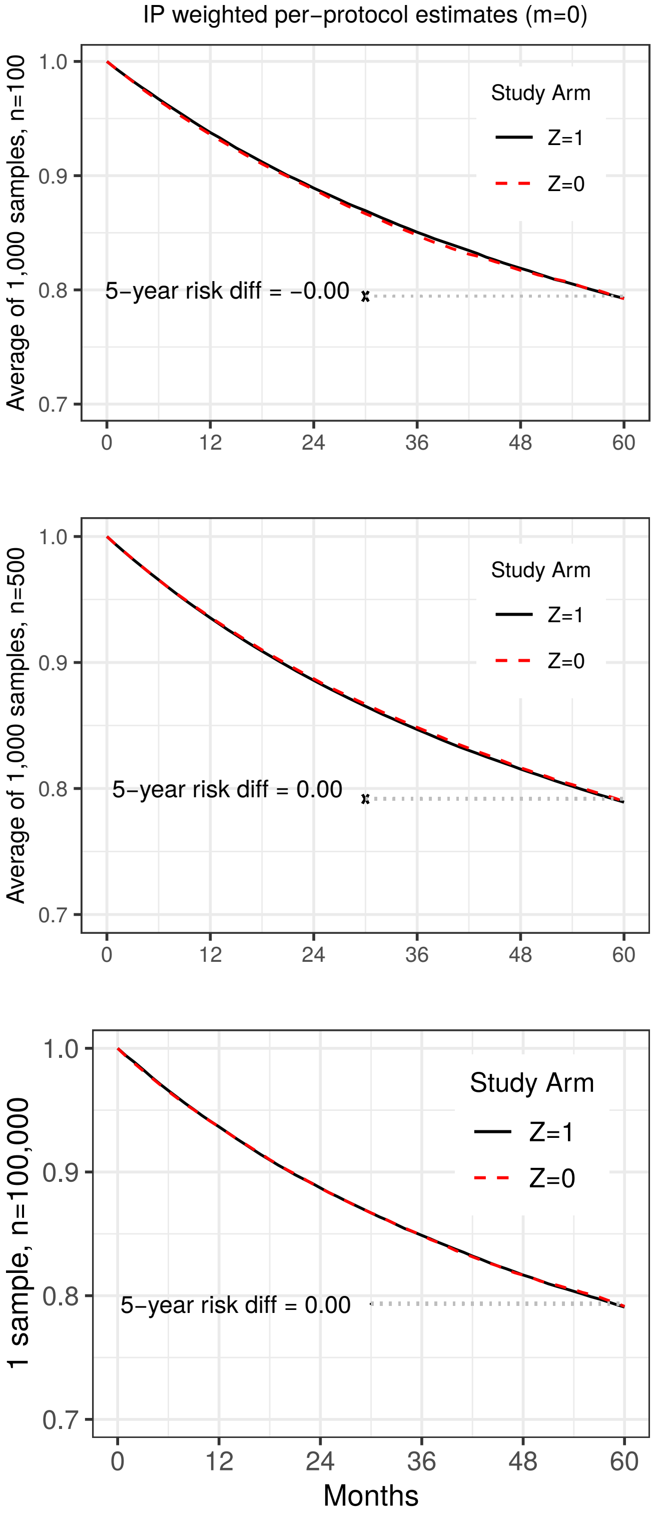

Figure 3: IP weighted per-protocol survival estimates by treatment arm under full measurement ($m = 0$). Comparison of bias calculations using a single large sample compared to an average of many smaller samples

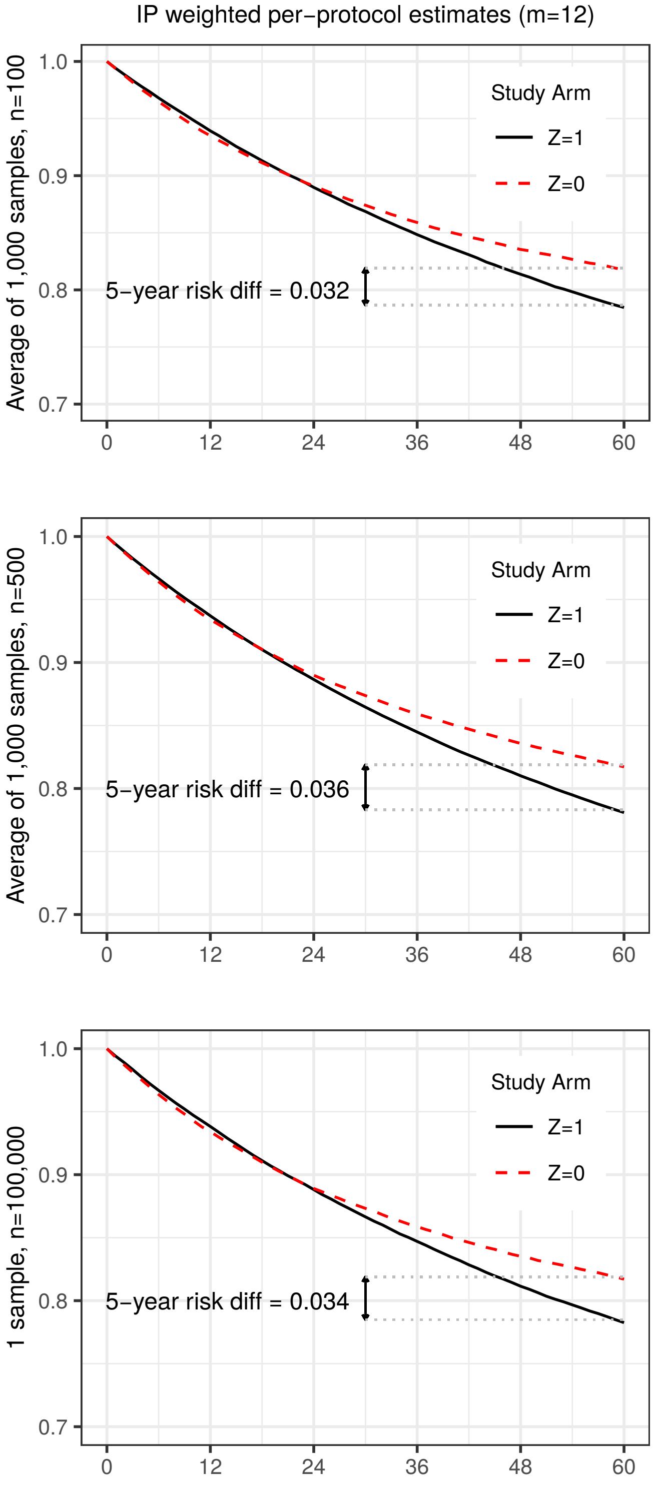

Figure 4: IP weighted per-protocol survival estimates by treatment arm under yearly measurement ($m = 12$). Comparison of bias calculations using a single large sample compared to an average of many smaller samples
